# Author Correction: Distinct local and global functions of mouse Aβ low-threshold mechanoreceptors in mechanical nociception

**DOI:** 10.1038/s41467-024-48095-6

**Published:** 2024-05-01

**Authors:** Mayank Gautam, Akihiro Yamada, Ayaka I. Yamada, Qinxue Wu, Kim Kridsada, Jennifer Ling, Huasheng Yu, Peter Dong, Minghong Ma, Jianguo Gu, Wenqin Luo

**Affiliations:** 1 Department of Neuroscience, Perelman School of Medicine, University of Pennsylvania, Philadelphia, PA 19104 USA; 2 Department of Anesthesiology and Perioperative Medicine, School of Medicine, University of Alabama at Birmingham, Birmingham, AL 35294 USA

**Keywords:** Pain, Sensory processing

Correction to: *Nature Communications* 10.1038/s41467-024-47245-0, published online 04 April 2024

In this article, the affiliation details in the Methods Section for Dr. Martyn Goulding were incorrectly given as ‘Scripp’s institute’ but should have been ‘Salk Institute’. The original article has been corrected.

