# Supplementary material for: Distinct local and global functions of mouse Aβ low-threshold mechanoreceptors in mechanical nociception
Source: Nat Commun. 2024 Apr 4;15:2911. doi: 10.1038/s41467-024-47245-0 (PMC10995180; doi:10.1038/s41467-024-47245-0)
Supplement: Supplementary file 1 — Supplementary Information [file 41467_2024_47245_MOESM1_ESM.pdf]

Supplementary Information – Table of Contents

1. Supplementary Tables

Supplementary Table 1, related to Figure 3: Summary of different thermal assays with control and ablated *Split^{Cre}-Aβ TauDTR* mice.

Supplementary Table 2, related to Figure 4: Summary of peripheral light-evoked behavioral responses of *Split^{Cre}-Aβ ReaChR* mice.

Supplementary Table 3, related to Figure 6: Summary of spinal light-evoked behavioral responses of *Split^{Cre}-Aβ ReaChR* mice.

2. Supplementary Figures

Supplementary Fig. 1, related to Figure 1: Histological characterizations of *Split^{Cre}-Aβ ReaChR* mice.

Supplementary Fig. 2, related to Figure 3: Histological characterization of *Split^{Cre}-Aβ TauDTR* mice.

Supplementary Fig. 3, related to Figure 4: Ablation of Split^{Cre}-Aβ-LTMRs did not alter general locomotion and thermal sensation.

Supplementary Fig. 4, related to Figure 4: Ablation of Split^{Cre}-Aβ-LTMRs altered the gentle touch and nociception in the hairy skin.

Supplementary Fig. 5, related to Figure 5: Local optogenetic activation of Split^{Cre}-Aβ-LTMRs at different skin areas, and CFA-induced inflammation altered the GFP^{+} fiber innervation in plantar skin.

Supplementary Fig. 6, related to Figure 7: Dorsal column activation of Split^{Cre}- ReaChR^{+} Aβ-LTMRs activated spinal cord dorsal horn neurons.

Supplementary Tables

Supplementary Table 1. Summary of different thermal assays in *Split*^{Cre}-*Aβ TauDTR* mice, related to Figure 4.

**Hargreaves**			
**Vehicle**	9.89 ± 0.47 s		
**DTA**	10.29 ± 0.75 s		
**Dry-ice**			
**Vehicle**	2.77 ± 0.05 s		
**DTA**	2.65 ± 0.08 s		
**Dynamic hotplate**			
**Vehicle**	49.4 ± 0.24℃		
**DTA**	49.73 ± 0.30℃		
**Static hotplate**			
	**Flick**	**Lick**	**Jump**
**Vehicle**	10.33 ± 0.81 s	22.01 ± 2.23 s	29.33 ± 0.58 s
**DTA**	9.79 ± 0.77 s	21.34 ± 2.47 s	29.83 ± 0.17 s
**Tail-immersion**			
	**48℃**	**50℃**	**55℃**
**Vehicle**	1.91 ± 0.07 s	1.26 ± 0.03 s	0.71 ± 0.02 s
**DTA**	1.83 ± 0.05 s	1.33 ± 0.04 s	0.72 ± 0.02 s

Supplementary Table 2. Summary of peripheral light-evoked behavioral response in *Split*^{Cre}*-Aβ ReaChR* mice, related to Figure 5.

Baseline						
	**5 mW**		**10 mW**		**20 mW**	
	**Response (%)**	**Latency (s)**	**Response (%)**	**Latency (s)**	**Response (%)**	**Latency (s)**
**Hind paw**	37.5 ± 13.27	4.01 ± 0.38	83.33 ± 8.9	2.76 ± 0.38	95.83 ± 4.17	1.88 ± 0.22
**Tail**	83.33 ± 8.9	2.82 ± 0.42	91.67 ± 5.45	1.78 ± 0.24	95.83 ± 4.17	0.78 ± 0.16

CFA-induced chronic inflammatory pain condition			
**5 mW**			
**Time post-CFA**	**Paw withdrawal frequency (%)**	**Latency (s)**	**Pain-score**
**Baseline**	37.03 ± 11.71	4.01 ± 0.38	0.04 ± 0.04
**2h**	74.07 ± 10.80	3.09 ± 0.31	1.11 ± 0.26
**D1**	88.89 ± 7.86	2.73 ± 0.33	1.79 ± 0.30
**D3**	81.48 ± 8.07	2.20 ± 0.19	1.33 ± 0.34
**D7**	88.89 ± 7.86	2.07 ± 0.21	2.41 ± 0.40
**D14**	96.30 ± 3.70	1.72 ± 0.27	1.96 ± 0.39
**D21**	88.89 ± 7.86	2.80 ± 0.19	1.18 ± 0.27
**D28**	92.59 ± 4.90	3.00 ± 0.39	1.15 ± 0.18

MPNL-induced chronic neuropathic pain condition			
**5 mW**			
**Time post-MPNL**	**Paw withdrawal frequency (%)**	**Latency (s)**	**Pain-score**
**Baseline**	33.17 ± 12.57	3.44 ± 0.58	0
**D1**	70.83 ± 11.68	2.83 ± 0.35	0.95 ± 0.20
**D3**	91.67 ± 5.46	2.33 ± 0.29	1.62 ± 0.22
**D7**	95.83 ± 4.17	1.87 ± 0.29	1.54 ± 0.21
**D14**	100 ± 0	1.54 ± 0.27	1.59 ± 0.27
**D21**	95.83 ± 4.17	1.50 ± 0.18	1.79 ± 0.26
**D28**	95.83 ± 4.17	1.48 ± 0.14	1.54 ± 0.14

Supplementary Table 3. Summary of spinal light-evoked behavioral responses in *Split^{Cre}-Aβ ReaChR* mice, related to Figure 7.

50% Paw withdrawal threshold (g)					
**Intensity (mW)**	**Pre-stim.**	**5 min.**	**30 min.**	**90 min.**	**120 min.**
**0.5**	0.985 ± 0.007	0.924 ± 0.017	0.953 ± 0.018	0.989 ± 0.007	0.986 ± 0.004
**10**	0.98 ± 0.009	0.405 ± 0.13	0.657 ± 0.064	0.88 ± 0.04	0.92 ± 0.02

	**Behaviors during light stimuli (15-minute period)**					
**Intensity (mW)**	**Back attending episodes**		**Hind paw licking episodes**		**Back scratching bouts**	
	**Pre-stim.**	**Stim.**	**Pre-stim.**	**Stim.**	**Pre-stim.**	**Stim.**
**0.5**	12.01 ± 4.35	38.0 ± 9.05	1.14 ± 0.40	1.87 ± 0.55	7.14 ± 1.72	12.71 ± 3.67

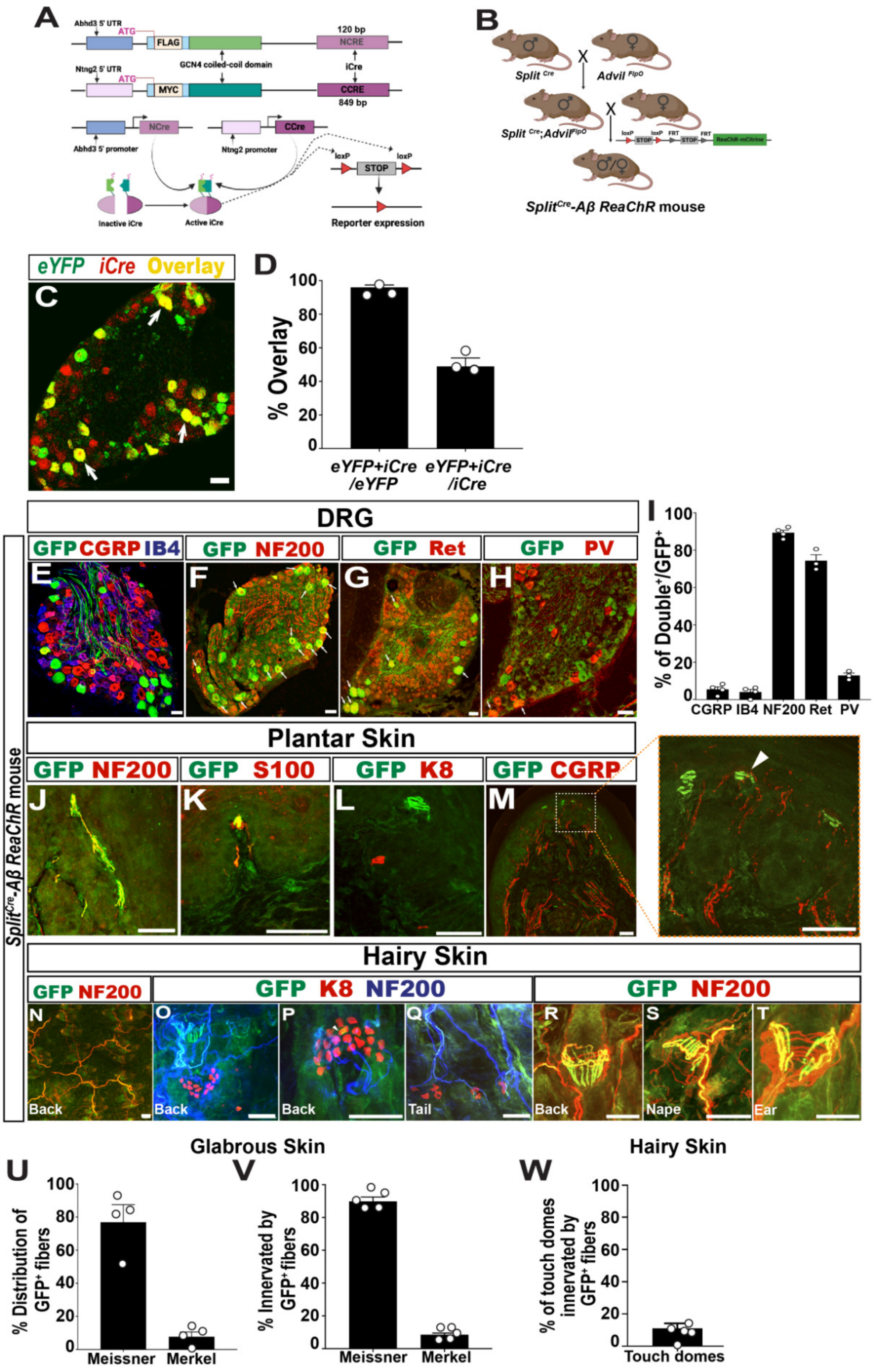

Supplementary Figure 1. Histological characterizations of *Split^{Cre}-Aβ ReaChR* mice.

(A) Schematic depicting the genetic configuration of the *Split*^{Cre} mouse line. (B) Breeding strategy to generate *Split*^{Cre}*;Advil*^{FlpO}*;Rosa*$^{ReaChRf/+}$ mice. (C) RNAScope of *iCre* and *Eyfp* with a lumbar DRG section. (D) Quantification. 96% of all double positive DRG neurons were iCre^{+}, whereas 48.9% of DRG neurons expressing *iCre* C terminal were Eyfp^{+}. (E) Immunostaining of a lumbar DRG section showing little overlap between GFP (ReaChR)$^{+}$ DRG neurons and nociceptor markers CGRP or IB4. (F & G) Immunostaining of DRG sections showing high overlaps between GFP (ReaChR)$^{+}$ DRG neurons and NF200 or RET. White arrows indicate some overlapped neurons. (H) Immunostaining of a DRG section showing some overlaps between GFP (ReaChR)$^{+}$ DRG neurons and a proprioceptive marker PV. (I) Quantification of the percentage of GFP^{+} neurons expressing different markers. (J-M) Images of dermal papilla sections of the plantar skin showing GFP^{+} nerves are NF200^{+} and innervate S100^{+} Meissner's corpuscles. GFP^{+} fibers rarely innervate the K8^{+} Merkel cell at the base of papilla. A few CGRP^{+} fibers also innervate some of the Meissner's corpuscles and skin regions around, but CGRP+ fibers and GFP+ fibers were non-overlapping. (N) Whole-mount (WM) immunostaining of the hairy back skin showing that GFP^{+} peripheral axons are NF200^{+}. (O-Q) WM immunostaining of the hairy back and tail skin showing that GFP^{+} peripheral axons are NF200^{+}. A few GFP^{+} fibers innervate Merkel cells in the hairy skin (an example indicated by a white arrowhead). (R-T) WM immunostaining of the hairy back, nape and posterior ear skin (U) Quantification of the percentage of GFP^{+} axons

innervating Meissner's corpuscles or Merkle cells in the glabrous skin sections. (V) Quantification of the percentage of Meissner's corpuscles or Merkle cells innervated by GFP+ axons in the glabrous skin sections. (W) Percentage of the touch domes innervated by GFP+ fibers in back hairy skin . Data are presented as Mean ± SEM. 6-8 sections/mouse, n = 4-6 mice except D (n = 3-4 sections/mouse, n = 3 mice). Scale bar represents 50 μm in all micrographs. Source data are provided as a Source Data file.

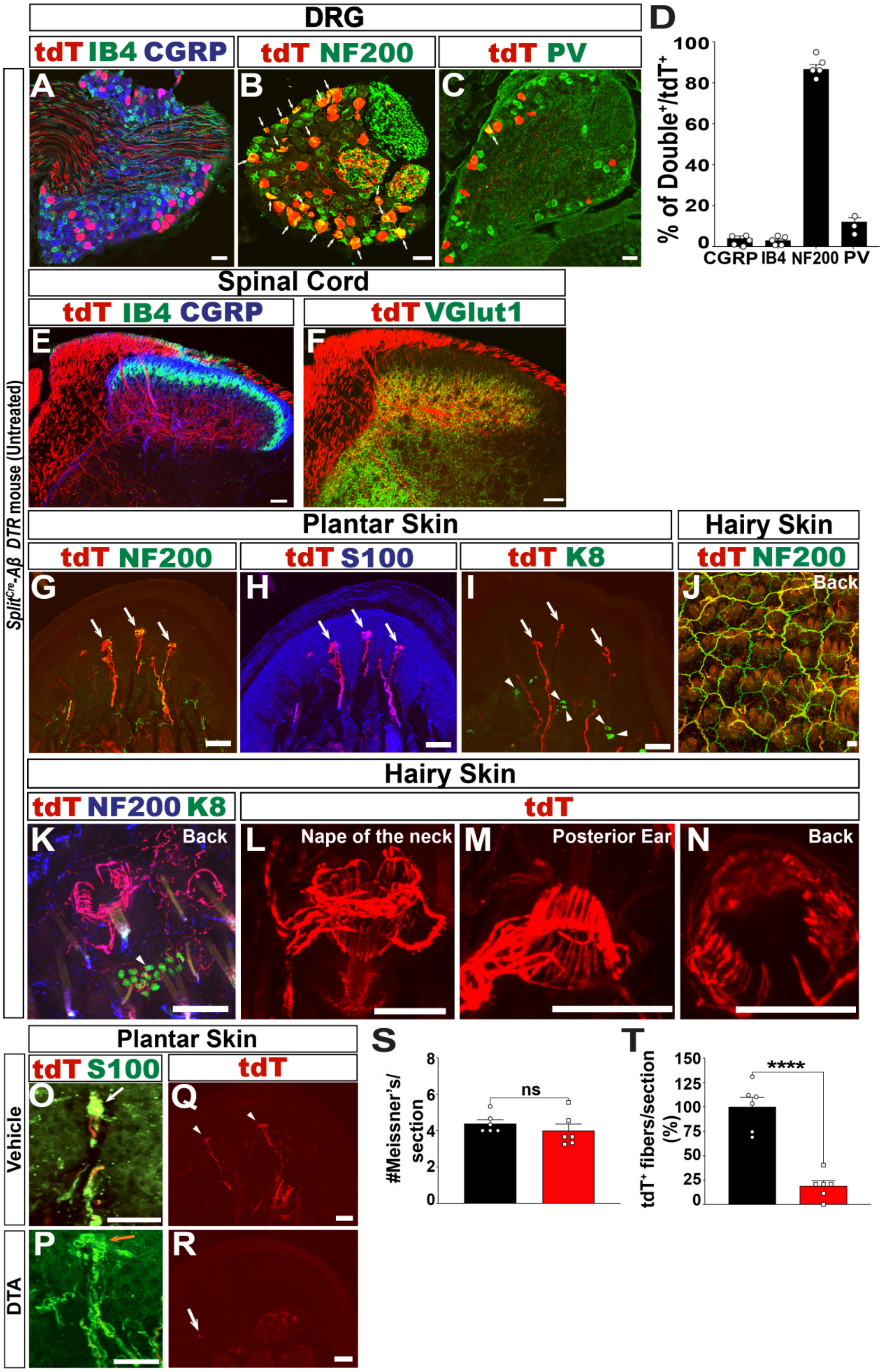

Supplementary Figure 2. Histological characterization of *Split^{Cre}-Aβ TauDTR* mice.

(A-C) Immunostaining of tdTomato with CGRP, IB4, NF200, or PV using DRG section of *SplitCre-Aβ TauDTR* mice. White arrows indicate some overlapped neurons. (D) Quantification of the percentage of tdTomato$^+$ neurons expressing different markers. 6-8 sections/mouse, n = 4-6 mice. (E-F) Immunostaining of a lumbar spinal cord section for tdTomato, CGRP, IB4 and VGLUT1. (G-I) Immunostaining of plantar skin sections for tdTomato, NF200, S100 or K8. White arrows and white arrowheads indicate some Meissner's corpuscles, and Merkel cells respectively. 6-8 sections/mouse, n = 4-6 mice. (J) Whole-mount (WM) immunostaining of the back skin shows that tdTomato$^+$ peripheral axons are NF200$^+$. (K) WM immunostaining of the back skin for tdTomato, NF200, and a few tdTomato fibers also innervate Merkel cells (Indicated by a white arrowhead). (L-N) WM immunostaining of the nape, posterior ear and back skin showing tdTomato$^+$ peripheral axons forming lanceolate endings. (O-P) Higher magnification images of dermal papillae sections showing DTA-induced ablation of tdTomato$^+$ afferents innervating in Meissner's corpuscles. White arrow indicates S100$^+$ Meissner's corpuscles with tdTom$^+$ fibers in a vehicle treated mouse, while red arrow shows a Meissner's corpuscle with loss of tdTom innervation in a DTA treated mouse. (Q-R) Images showing tdTomato$^+$ nerve fibers in the plantar skin sections of vehicle or DTA treated mice. White arrowheads indicate Meissner's corpuscle's like tdT$^+$ innervating fibers in vehicle-treated mice, and white arrow points to a remaining tdTomato$^+$ fiber in the dermis of DTA-treated mice. (S) Quantification of the number of Meissner's

corpuscles per footpad did not show significant alteration after ablation. (T) Quantification of the number of tdTomato$^+$ fibers innervating dermal papillae per footpad section showed a significant decrease with DTA treatment, $p = 0.0022$. The data were normalized to the average number of tdTomato$^+$ fibers innervating dermal papillae of control mice. 5-7 sections per mouse, $n = 6$ mice. Error bars represent Mean ± S.E.M. Unpaired, two-tailed Mann-Whitney test. ****$p < 0.0001$. Scale bar represents 50 μm in all micrographs. Source data are provided as a Source Data file.

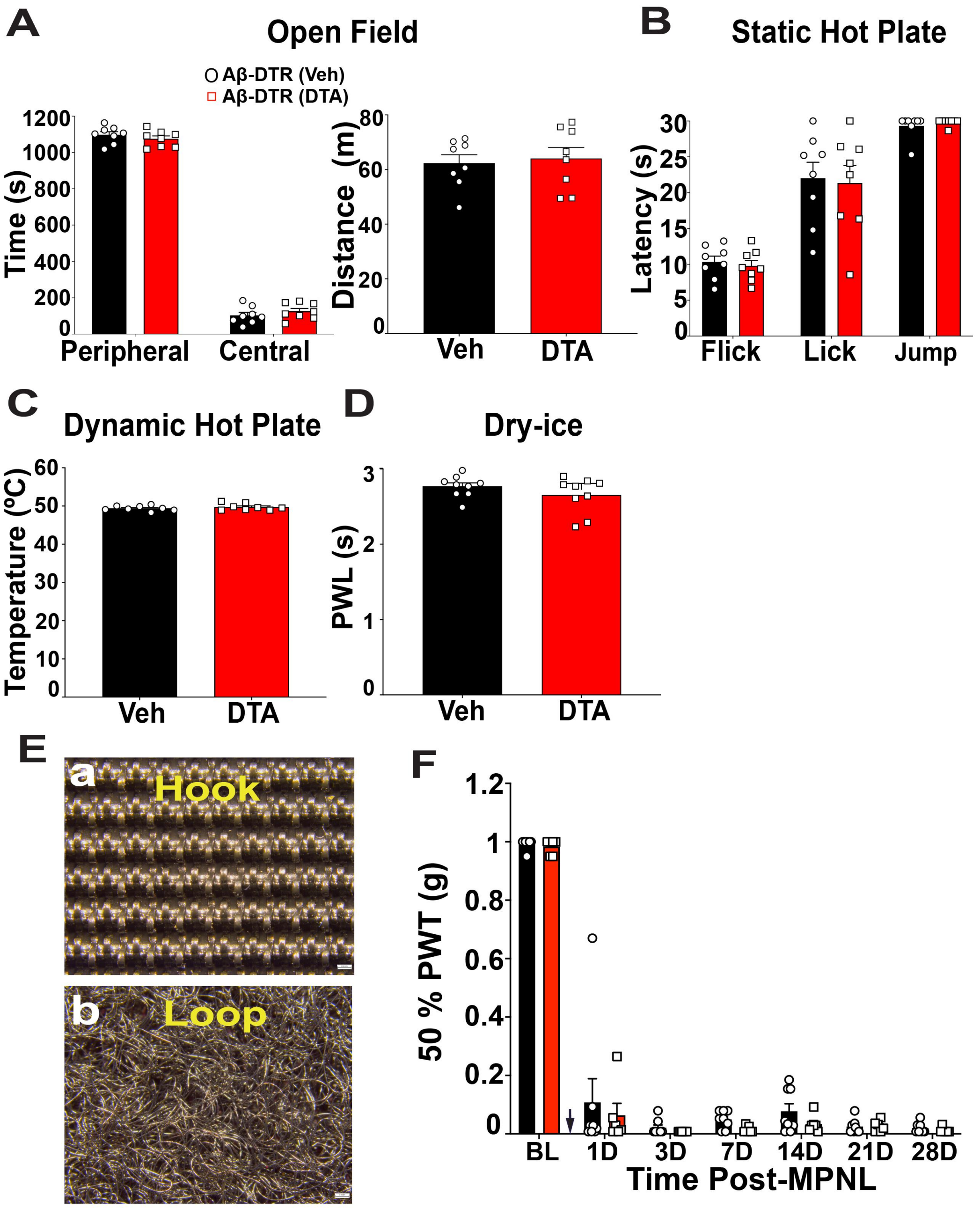

Supplementary Figure 3. Ablation of Split^{Cre}-Aβ-LTMRs did not alter general locomotion and thermal sensation.

(A) Ablation of $Split^{Cre}$-Aβ-LTMRs did not alter the time spent in peripheral ($p = 0.38$) and central zones ($p = 0.328$) in vehicle vs DTA treated mice. There was also no difference in total distance travelled between the two groups ($p = 0.72$). (B) No difference in baseline heat nociception sensitivity, indicated by the latency of hind paw flick ($p = 0.64$), hind paw lick ($p = 0.904$), or jump ($p = 0.733$), in the DTA vs vehicle treated groups in static hotplate test. (C) No difference in baseline heat nociception sensitivity, indicated by the heat pain temperature threshold ($p = 0.702$) between the DTA vs vehicle treated using the dynamic hotplate test, n = 8 mice in each group in (A-C). (D) No difference in baseline cold nociception sensitivity, indicated by the paw-withdrawal latency ($p = 0.395$) to dry ice, between the DTA vs vehicle treated groups, n = 9 mice in each group. (E) Representative images of (a) hook and (b) loop material of the Velcro surface, scale bar = 0.5 mm. (F) The ablated mice showed no difference in changes of 50% PWT from the vehicle control group (vehicle: n = 8 mice and DTA: n = 6 mice), using a neuropathic median plantar nerve ligation model. Black arrow indicates the time of medial plantar nerve ligation. Black and red color of the bars represent vehicle and DTA treatment respectively. Error bars represent Mean ± S.E.M. Unpaired, two-tailed Mann-Whitney test. $^*p < 0.05$, $^{**}p < 0.01$; $^{***}p < 0.001$, ns = non significance, BL = baseline. Source data are provided as a Source Data file.

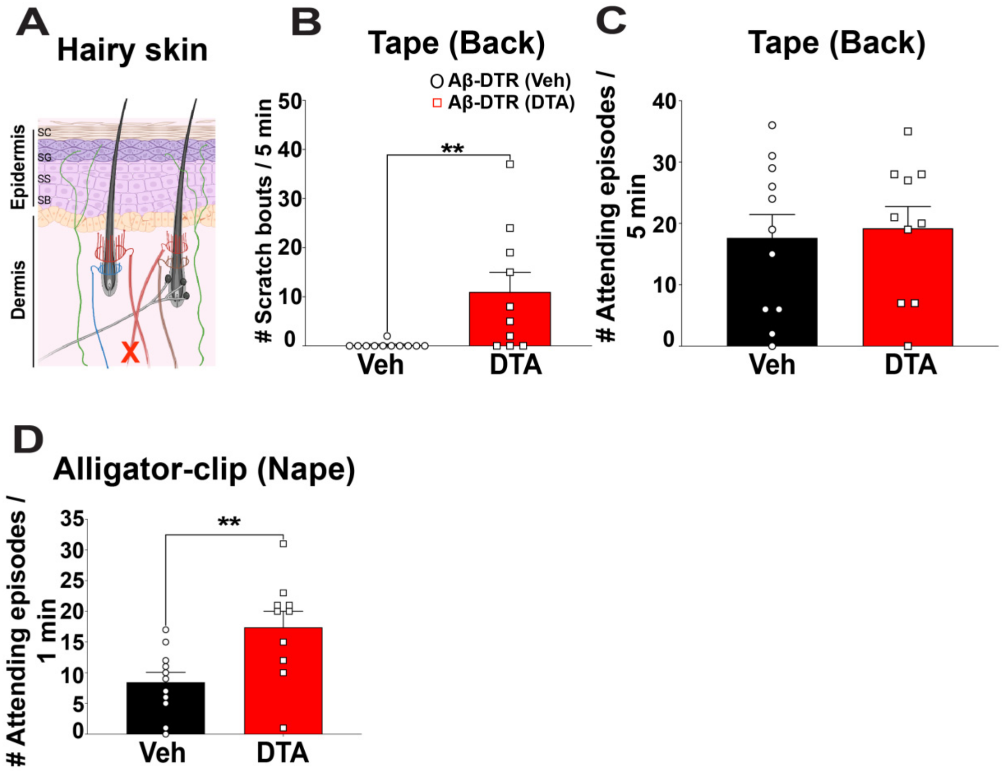

Supplementary Figure 4. Ablation of Split^{Cre}-Aβ-LTMRs altered the gentle touch and mechanical nociception in the hairy skin.

(A) Illustration showing cutaneous sensory afferents of the ablated hairy skin; green fibers represent nociceptive C/Aδ fibers; red fibers represent RA Aβ-LTMRs encircling the hair follicles; black fibers represent SA Aβ-LTMRs innervating Merkel cells (black bead-like structures); blue and brown colored fibers represent the C- and Aδ-LTMRs respectively; 'X' symbol represents the DTA-induced selective ablation of $Split^{Cre}$-Aβ-LTMRs. (B) The ablated mice showed significantly increased scratching behaviors in response to a tape attached to the back ($p = 0.0027$). (C) In response to a tape attached to the back, no difference in attending behaviors between the vehicle and DTA groups ($p = 0.743$) was observed. (D) The ablated mice displayed significantly increased number of licking episodes ($p = 0.0074$) in response to the application of an alligator clip at the neck nape. n = 11 and 10 mice in vehicle and DTA groups respectively in (B-D). Also see the supplementary movie 3. Error bars represent Mean ± S.E.M. Unpaired, two-tailed Mann-Whitney test. **$p < 0.01$. Source data are provided as a Source Data file.

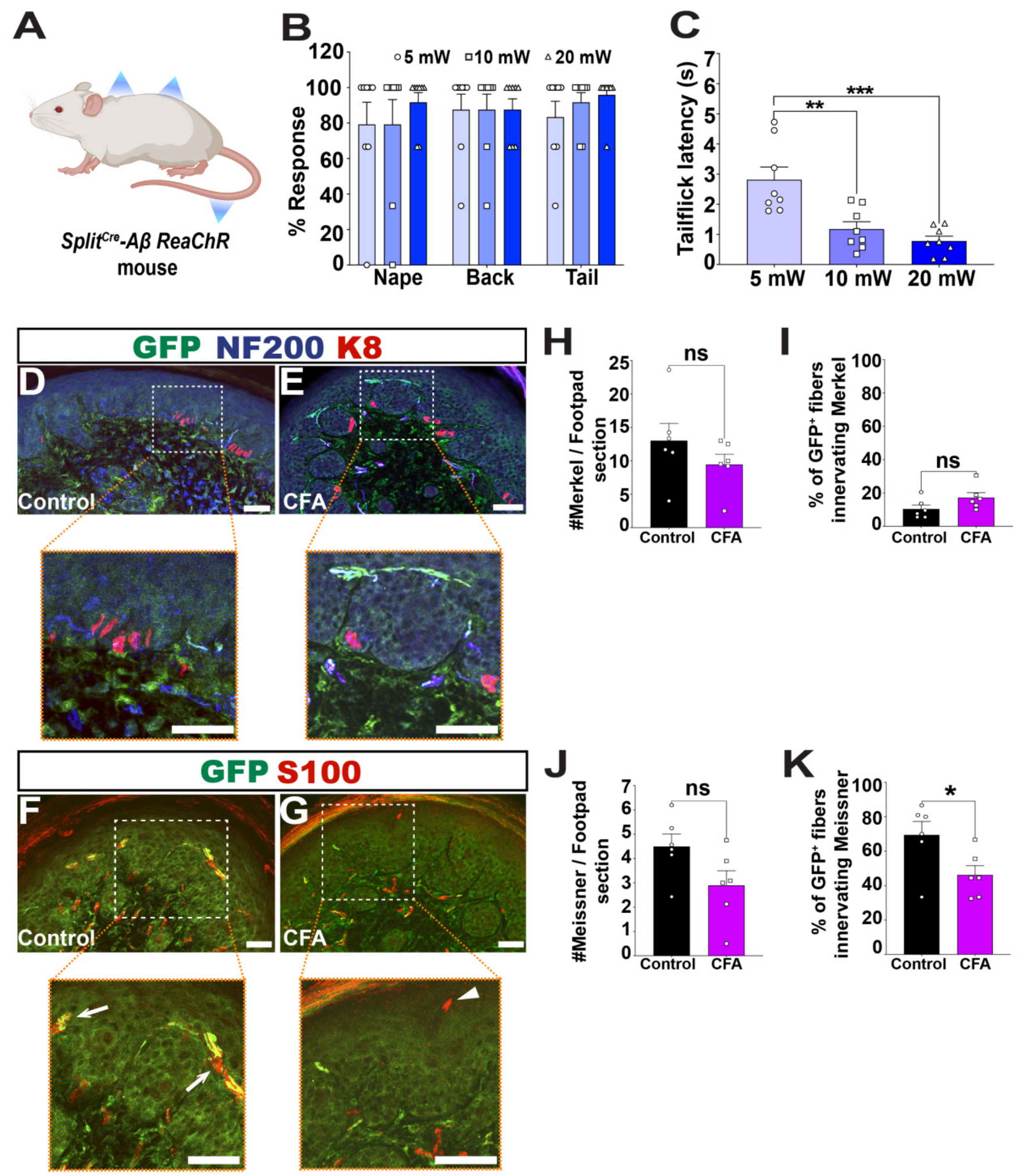

Supplementary Figure 5. Local optogenetic activation of Split^{Cre}-Aβ-LTMRs at different skin areas, and CFA-induced inflammation alter the GFP^{+} fiber innervation in plantar skin.

(A) Schematic of blue laser stimulation on different skin areas of the *Split*^{Cre}*-Aβ ReaChR* mouse. (B) *M*ice showed high responsive rates to different intensities (5, 10 and 20 mW) of blue laser stimuli on different hairy skin (Nape of the neck, back and tail) regions. (C) The tail-flick latency decreased significantly in an intensity dependent manner (5 mW vs 10 mW, $p = 0.005$); (5 mW vs 20 mW, $p = 0.0008$); (10 mW vs 20 mW, $p = 0.68$). n = 8 mice in (B-C). (D-E) Immunostaining of GFP, NF200 and K8 with plantar footpad sections (low and high magnifications) of control and CFA-treated *Split*^{Cre}*-Aβ ReaChR* mice at post-CFA Day 7. (F-G) Immunostaining of GFP and S100 with footpad sections (low and high magnifications) of control and CFA-treated *Split*^{Cre}*-Aβ ReaChR* mouse at post-CFA Day 7. S100$^+$ Meissner corpuscles innervated or not innervated by GFP+ fibers are indicated by yellow and red arrows, respectively. (H) Merkel cell numbers per foot pad skin section showed no significant difference between the control and CFA treated mice ($p = 0.179$) at post-CFA Day 7. (I) Percentage of Merkel cells innervated by GFP$^+$ fibers per foot pad section showed a non-significant increase in CFA treated mice, compared to the control ($p = 0.088$). (J) Number of Meissner's corpuscles per foot pad section showed non-significant decrease trend in control and CFA-treated mice ($p = 0.0931$). (K) Percentage of Meissner's corpuscles innervated by GFP$^+$ fibers per foot pad section showed a significant decrease in CFA treated mice compared to the control ($p = 0.041$), n = 6 mice in each group (5-7 sections per mouse). Error bars represent Mean ± S.E.M. One way ANOVA followed by

Holm-Šídák's multiple comparisons test in C while two-sided Mann-Whitney test in H-K. *$p < 0.05$, **$p < 0.01$, ***$p < 0.001$, ****$p < 0.0001$. Scale bar represents 50 μm in all micrographs. Source data are provided as a Source Data file.

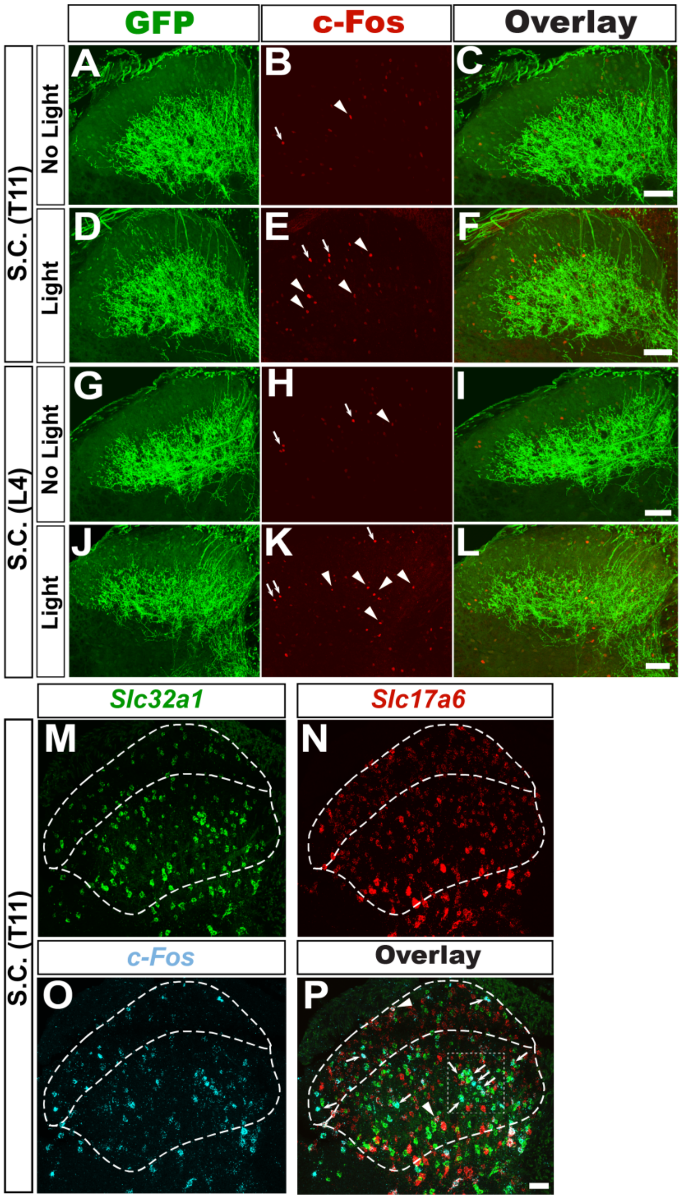

Supplementary Figure 6. Dorsal column activation of Split^{Cre}-ReaChR^{+} Aβ-LTMRs evoked spontaneous behaviors and c-Fos expression in deep dorsal horn neurons.

(A-C) Double immunostaining of c-Fos and GFP with a T11 spinal cord section of an unstimulated *Split^{Cre}-Aβ ReaChR* mouse showing innervation of ReaChR-EFYP$^+$ central terminals in the layer III-V and baseline c-Fos expression. (D-F) Double immunostaining of c-Fos and GFP with a T11 spinal cord section of a light-stimulated *Split^{Cre}-Aβ ReaChR* mouse showing innervation of ReaChR-EFYP$^+$ central terminals in the layer III-V and increased c-Fos expression. (G-I) Double immunostaining of c-Fos and GFP with a L4 spinal cord section of an unstimulated *Split^{Cre}-Aβ ReaChR* mouse showing innervation of ReaChR-EFYP$^+$ central terminals in the layer III-V and baseline c-Fos expression. (J-L) Double immunostaining of c-Fos and GFP with a L4 spinal cord section of a light-stimulated *Split^{Cre}-Aβ ReaChR* mouse showing innervation of ReaChR-EFYP$^+$ central terminals in the layer III-V and increased c-Fos expression. White arrowheads indicate some examples of c-Fos immunoreactivity in deeper laminae. White arrows point to some c-Fos$^+$ cells in superficial laminae of the dorsal horn. n = 3 mice in each group (5-7 sections per mouse for statistical comparison). (M-P) Representative low-magfication images of RNAScope *in situ* hybridization for *Slc32a1*, *Slc17a6,* and *c-Fos* with a T11 spinal cord section of a spinal light-stimulated *Split^{Cre}-Aβ ReaChR* mouse. White arrows indicate some co-localized Slc32a1$^+$ with c-Fos$^+$ neurons. White arrowheads indicate some examples of co-localized Slc17a6$^+$ and c-Fos$^+$ neurons. High magnification images and quantification are shown in the Fig. 6D-E. n = 3 mice (3-4 sections per mouse). Scale bar represents 50 μm in all micrographs.
