# Supplementary material for: Distinct local and global functions of mouse Aβ low-threshold mechanoreceptors in mechanical nociception
Source: Nat Commun. 2024 Apr 4;15:2911. doi: 10.1038/s41467-024-47245-0 (PMC10995180; doi:10.1038/s41467-024-47245-0)
Supplement: Supplementary file 3 — Description of Additional Supplementary Files [file 41467_2024_47245_MOESM3_ESM.pdf]

DESCRIPTION OF ADDITIONAL SUPPLEMENTARY FILES DOCUMENT

Supplementary Movie 1: Tape removal experiment. Movie showing behaviors of vehicle- (control) or DTA-treated $Split^{Cre}$-Aβ TauDTR mice in response to a tape sticking to one hind paw.

Supplementary Movie 2: Mechanical nociception (alligator) at the plantar skin. Movie showing behaviors of vehicle- (control) or DTA-treated $Split^{Cre}$-Aβ TauDTR mice in response to an alligator clip applied to one hind paw.

Supplementary Movie 3: Mechanical nociception (alligator) at the neck nape. Movie showing behaviors of vehicle-(control) or DTA-treated $Split^{Cre}$-Aβ TauDTR mice in response to an alligator clip applied at the neck nape.

Supplementary Movie 4: Peripheral optogenetic experiment of $Split^{Cre}$-Aβ ReaChR mice at the baseline. High-speed imaging video showing the touch-like responses/reflex of a saline-treated $Split^{Cre}$-AβReaChR mouse in response to 5 mW blue laser stimuli at the paw plantar region.

Supplementary Movie 5: Peripheral optogenetic response of $Split^{Cre}$-Aβ ReaChR mice at post-CFA day 7. High-speed imaging video showing the nocifensive responses/reflex of a $Split^{Cre}$-Aβ ReaChR mouse, 7 days after CFA-treatment, in response to 5 mW blue laser stimuli at the inflammatory paw plantar region.

Supplementary Movie 6: Peripheral optogenetic experiment of $TrpV1^{Cre}$-ReaChR mice at the baseline. High-speed imaging video showing the nocifensive responses/reflex of an untreated $TrpV1^{Cre}$-ReaChR mouse in response to 5 mW blue laser stimuli at the paw plantar region.
