# Supplementary material for: Distinct local and global functions of mouse Aβ low-threshold mechanoreceptors in mechanical nociception
Source: Nat Commun. 2024 Apr 4;15:2911. doi: 10.1038/s41467-024-47245-0 (PMC10995180; doi:10.1038/s41467-024-47245-0)
Supplement: Supplementary file 10 — Reporting Summary [file 41467_2024_47245_MOESM10_ESM.pdf]

Corresponding author(s): Jianguo Gu, Wenqin Luo

Last updated by author(s): Jan 18, 2024

Reporting Summary

Nature Portfolio wishes to improve the reproducibility of the work that we publish. This form provides structure for consistency and transparency in reporting. For further information on Nature Portfolio policies, see our Editorial Policies and the Editorial Policy Checklist.

Statistics

For all statistical analyses, confirm that the following items are present in the figure legend, table legend, main text, or Methods section.

n/a	Confirmed	
☐	☒	The exact sample size (*n*) for each experimental group/condition, given as a discrete number and unit of measurement
☐	☒	A statement on whether measurements were taken from distinct samples or whether the same sample was measured repeatedly
☐	☒	The statistical test(s) used AND whether they are one- or two-sided<br>*Only common tests should be described solely by name; describe more complex techniques in the Methods section.*
☐	☒	A description of all covariates tested
☐	☒	A description of any assumptions or corrections, such as tests of normality and adjustment for multiple comparisons
☐	☒	A full description of the statistical parameters including central tendency (e.g. means) or other basic estimates (e.g. regression coefficient) AND variation (e.g. standard deviation) or associated estimates of uncertainty (e.g. confidence intervals)
☐	☒	For null hypothesis testing, the test statistic (e.g. *F*, *t*, *r*) with confidence intervals, effect sizes, degrees of freedom and *P* value noted<br>*Give P values as exact values whenever suitable.*
☒	☐	For Bayesian analysis, information on the choice of priors and Markov chain Monte Carlo settings
☒	☐	For hierarchical and complex designs, identification of the appropriate level for tests and full reporting of outcomes
☒	☐	Estimates of effect sizes (e.g. Cohen's *d*, Pearson's *r*), indicating how they were calculated

Our web collection on statistics for biologists contains articles on many of the points above.

Software and code

Policy information about availability of computer code

Data collection	The electrophysiological data were collected using Clampex 11 (Commercial software).<br>Immunofluorescence data were collected in LAS X Leica Microsystems.<br>High-speed imaging videos were collected on FASTCAM Viewer 4 (Photron).<br>Other data were collected in WPS and MS office.
Data analysis	Statistical tests and data analysis in Electrophysiology study were performed by Clampfit and Prism 8 (Commercial softwares).<br>Immunofluorescence and whole-mount imaging data were also analyzed by Fiji/ImageJ (v2.3.0;NIH).<br>Rest of the data were analyzed using Graph Pad Prism 9.

For manuscripts utilizing custom algorithms or software that are central to the research but not yet described in published literature, software must be made available to editors and reviewers. We strongly encourage code deposition in a community repository (e.g. GitHub). See the Nature Portfolio guidelines for submitting code & software for further information.

Data

Policy information about availability of data

All manuscripts must include a data availability statement. This statement should provide the following information, where applicable:

- Accession codes, unique identifiers, or web links for publicly available datasets
- A description of any restrictions on data availability
- For clinical datasets or third party data, please ensure that the statement adheres to our policy

All data needed to evaluate the conclusions in the paper are available in the paper and the supplementary information. The raw data for all main and Supplementary Figures are available in Source Data files, accompanying this paper. For information and requests related to electrophysiological recordings of this manuscript, please contact Dr. Jianguo Gu (jianguogu@uabmc.edu). For inquiries related to other results and experiments in this manuscript, please contact Dr. Wenqin Luo (luow@pennmedicine.upenn.edu).
Source data are provided with this paper.

Research involving human participants, their data, or biological material

Policy information about studies with human participants or human data. See also policy information about sex, gender (identity/presentation), and sexual orientation and race, ethnicity and racism.

Reporting on sex and gender	N/A
Reporting on race, ethnicity, or other socially relevant groupings	N/A
Population characteristics	N/A
Recruitment	N/A
Ethics oversight	N/A

Note that full information on the approval of the study protocol must also be provided in the manuscript.

Field-specific reporting

Please select the one below that is the best fit for your research. If you are not sure, read the appropriate sections before making your selection.

☒ Life sciences ☐ Behavioural & social sciences ☐ Ecological, evolutionary & environmental sciences

For a reference copy of the document with all sections, see nature.com/documents/nr-reporting-summary-flat.pdf

Life sciences study design

All studies must disclose on these points even when the disclosure is negative.

Sample size	Sample sizes for individual experiments followed the field traditions or were determined according to the NIH "Guidelines for the Care and Use of Mammals in Neuroscience and Behavioral Research" (Sample Size Determination: https://www.ncbi.nlm.nih.gov/books/NBK43321/).
Data exclusions	No data were excluded.
Replication	The number of replications for each experiment was included in the figure legends.
Randomization	Animals and recorded fibers were chosen randomly for in vitro electrophysiological experiments. Skin/DRG/spinal cord sections were randomly chosen for histological analysis.
Blinding	In vitro electrophysiological recordings were not performed in a manner blinded to genotype, as only triple mice expressing ReaChR2 were recorded, or treatment, due to the easily visible inflammatory phenotype of the CFA treated hind-paw. The experimenter was blind to the genotype of the mice while performing most of the behavioral experiments, but not to the chronic pain models (CFA-induced inflammatory pain or MPNL neuropathic pain) due to the easily visible inflammatory phenotype of the treated hind-paw and the paw guarding posture of the affected mice.

Reporting for specific materials, systems and methods

We require information from authors about some types of materials, experimental systems and methods used in many studies. Here, indicate whether each material, system or method listed is relevant to your study. If you are not sure if a list item applies to your research, read the appropriate section before selecting a response.

Materials & experimental systems

n/a	Involved in the study
☐	☒ Antibodies
☒	☐ Eukaryotic cell lines
☒	☐ Palaeontology and archaeology
☐	☒ Animals and other organisms
☒	☐ Clinical data
☒	☐ Dual use research of concern
☒	☐ Plants

Methods

n/a	Involved in the study
☒	☐ ChIP-seq
☒	☐ Flow cytometry
☒	☐ MRI-based neuroimaging

Antibodies

Antibodies used

Chicken polyclonal anti-GFP Aves Labs Cat# GFP-1020; RRID:AB_10000240
Rabbit polyclonal anti-GFP lnvitrogen Cat# A-11122; RRID:AB_221569
Rabbit polyclonal anti-CGRP lmmunoStar Cat# 24112; RRID:AB_572217
Rabbit polyclonal anti-NF200 Sigma-Aldrich Cat# N4142; RRID:AB_ 477272
Chicken polyclonal anti-NF200 Aves Labs Cat# NFH-3-1003; RRID:AB_2313552
Guinea pig polyclonal anti-VGLUTl Millipore Cat# AB5905; RRID:AB_2301751
Rabbit polyclonal anti-Sl00 Abeam Cat# ab34686; RRID:AB_777793
Rat anti-KS (Troma-1) Univ of lowa/DSHB RRID:N/A
Rabbit anti-Parvalbumin Swant Cat# PV27; RRID:AB_2631173
Rabbit monoclonal anti-Ret lmmuno-Biological Laboratories Cat# 18121; RRID:AB_2301042
Rabbit polyclonal anti-DsRed Takara Bio Cat# 632496; RRID:AB_10013483
Rabbit monoclonal anti-c-Fos Cell Signaling Technology Cat# 2250; RRID:AB_2247211
IB4, Alexa Fluor 488 conjugate lnvitrogen Cat# 121411; RRID:AB_2314662
IB4, Alexa Fluor 594 conjugate lnvitrogen Cat# 121413; RRID:AB_2313921
Goat anti-rabbit lgG (H+L), Alexa Fluor 488 Thermo Fisher Scientific Cat# A-11008; RRID:AB_143165
Goat anti-rabbit lgG (H+L), Alexa Fluor 594Thermo Fisher Scientific Cat# A-11012; RRID:AB_2534079
Goat anti-rabbit lgG (H+L), Alexa Fluor 647 Thermo Fisher Scientific Cat# A-21244; RRID:AB_2535812
Goat anti-rabbit Cy3 Thermo Fisher Scientific Cat# A10520, RRID:AB_2534029

Validation

Validations from the manufacturer's websites.

Animals and other research organisms

Policy information about studies involving animals; ARRIVE guidelines recommended for reporting animal research, and Sex and Gender in Research

Laboratory animals

Animals were housed in facilities at the University of Pennsylvania and at the University of Alabama at Birmingham. Adult male and female mice of desired genotypes were kept in a standard 12-h light/dark cycle at controlled room temperature (20-23°C) and a humidity level between 30-70 %, with water and food pellets available ad libitum.
In electrophysiology experiments, SplitCre;AdvilFlpO;RosaReaChRf/+ mice (31 mice) were 6 to 15 weeks old and 4 were over 20 weeks old. In rest of all experiments, mice of age between 6-24 weeks were used. Following is the information of the mice used in the study:
1. AdvillinFlpO mice from Dr. David Ginty
2. SplitCre mice from Dr. David Ginty
3. RosaReaChR mice from Jackson Laboratory (USA) JAX: #024846
4. Tauds-DTRf/f mice from Dr. Martyn Goulding
5. Ai9tdTomatof/f mice from Jackson Laboratory (USA) JAX: #007905
6. C57BL/6J mice from Jackson Laboratory (USA) JAX: #000664
7. TrpVlCre mice from Jackson Laboratory (USA) JAX: #017769

Wild animals

No wild animals were used in the study.

Reporting on sex

Both male and female mice were used in all experiments and they were separately analyzed initially. Since no sex difference was evident, in later experiments, the data from both sexes were pooled.

Field-collected samples

No field collected samples were used in the study.

Ethics oversight

All experimental procedures were performed in accordance with the guidelines of the National Institutes of Health and were approved by the Institutional Animal Care and Use Committees at the University of Pennsylvania and the University of Alabama at Birmingham.

Note that full information on the approval of the study protocol must also be provided in the manuscript.

Plants

Seed stocks	N/A
Novel plant genotypes	N/A
Authentication	N/A
